# The Diagnosis of Albright’s Osteodystrophy in a Case With Respiratory Failure

**DOI:** 10.7759/cureus.55200

**Published:** 2024-02-29

**Authors:** Rodrigo Rufino, Daniela Rodrigues, Inês Carvalho, Patrícia Ferreira, Marta Carinhas

**Affiliations:** 1 Internal Medicine, Centro Hospitalar Barreiro-Montijo, Barreiro, PRT

**Keywords:** albright’s osteodystrophy, hypocalcemia, gnas mutation, respiratory failure, pseudohypoparathyroidism

## Abstract

Albright’s hereditary osteodystrophy is a rare hereditary disease due to a mutation of the complex guanine nucleotide-binding protein, alpha-stimulating activity polypeptide. This condition is commonly associated with type 1A and 1C pseudohypoparathyroidism and pseudo-pseudohypoparathyroidism due to resistance of parathyroid hormone. Patients present with specific characteristics such as brachydactyly, short stature, round facies, subcutaneous ossifications, developmental delay, and obesity, associated with hypocalcemia and hyperphosphatemia. This case presents a 55-year-old woman with short stature and neurocognitive impairment, who was admitted to the emergency department with acute decompensated heart and respiratory failure. On admission, hypocalcemia and hyperphosphatemia were noted, which in combination with the patient’s clinical history led to an etiological investigation. This case stresses the importance of not only treating the acute disease but also looking at the patient and their clinical and analytical features to diagnose this disease and prevent its complications.

## Introduction

Fuller Albright first described the phenotype of Albright’s hereditary osteodystrophy (AHO) in 1942 [1,2]. It is caused by mutations in the complex GNAS locus on the 20q13.3 chromosome [3,4]. When a mutated maternal GNAS1 allele is present, the disease is fully expressed causing pseudohypoparathyroidism (PHP), while paternal allele mutation causes only partial expression, known as pseudo-pseudohypoparathyroidism (PPHP) [5]. PHP is a group of disorders defined by parathyroid hormone (PTH) resistance at the kidney and bone [6]. PTH influences bone remodeling through stimulating calcium and bone reabsorption at the renal tubular [7]. It also converts calcidiol (25-hydroxyvitamin D) to calcitriol in the renal tubular cells, providing calcium absorption at the intestine and bone turnover [7]. PTH also reduces the reabsorption of phosphate mostly at the renal proximal tubule [8].

AHO is rare and patients usually present with hypocalcemia and hyperphosphatemia in association with elevated plasma PTH levels [5]. Clinical features include brachydactyly, short stature, round facies, subcutaneous ossifications, developmental delay, and obesity [1,3,4]. A provisional diagnosis can be implied when clinical criteria or other associated signs of hormonal resistance are present [4].

Here, we report the case of a 55-year-old woman with short stature and neurocognitive impairment who presented to the emergency department (ED) with signs of decompensated heart failure and associated ionic changes (severe hypocalcemia and hypokalemia, hypomagnesemia, and hyperphosphatemia). Once the acute disease and respiratory failure were resolved, the patient analytically presented subclinical hypothyroidism, which led to the etiological study and confirmation of PHP.

## Case presentation

We describe the case of a 55-year-old woman with a history of short stature and neurocognitive impairment but without any diagnosis and no other clinical or familial history. She lived with her brother and was entirely dependent. She presented to the ED with dyspnea and increased abdominal perimeter with a one-week onset. On admission, she was tachypneic with peripheral saturation of 87-89% in air, pulmonary auscultation with bilateral crackles, a sore abdomen on palpation of the left quadrants, and bilateral peripheral edema. The abnormal laboratory findings on admission are presented in Table 1.

**Table 1:** Laboratory findings on admission to the emergency department.

Laboratory test	Patient value	Normal value
Calcium	5.2 mg/dL	8.4–10.2 mg/dL
Phosphorus	7.4 mg/dL	2.3–4.7 mg/dL
Magnesium	1.5 mg/dL	1.59–2.56 mg/dL
Albumin	3.7 g/dL	3.5–5.2 mg/dL
Thyroid-stimulating hormone	2.8 µUI/mL	0.36–4.94 µUI/mL
Serum-free thyroxine	1.03 ng/mL	0.7–1.48 ng/mL

Arterial blood gas analysis showed global respiratory insufficiency with slight acidemia (pH: 7.330, pCO_2_: 68.6 mmHg, pO_2_: 53.4 mmHg, saturation of 85.8%, normal lactate and bicarbonate). The patient underwent computed tomography with no significant changes identified. Acute decompensated heart failure with respiratory insufficiency was assumed in the patient with probable sleep apnea obesity syndrome, associated with severe hypocalcemia, hypomagnesemia, and hyperphosphatemia. The patient started non-invasive mechanical ventilation and was admitted to the internal medicine department.

During hospitalization, the patient evolved favorably, with the resolution of respiratory insufficiency and correction of initial ionic changes, but now presented with subclinical hypothyroidism (thyroid-stimulating hormone (TSH) 6.30 µUI/mL and serum-free thyroxine (T4) 0.8 ng/mL). Due to the patient’s clinical features along with the analytical changes, the suspicion of PHP arose.

The patient was discharged a week and a half after admission. She was fully compensated, oriented to internal medicine consultation with ion oral supplementation, and was awaiting *GNAS1* mutation and PTH results. The genetic test revealed the variant of unknown clinical significance c.989A>G p.(Glu330Gly) in heterozygosity in the *GNAS *gene, with a normal PTH (48.2 ng/L) value.

## Discussion

Skeletal abnormalities, namely, the shortening of all long bones, are one of the most common features seen in 80% of patients [1]. Patients with type 1A PHP may also present with hearing loss, decreased olfaction, sleep apnea, and asthma-like symptoms [4]. The initial study should include serum calcium, albumin, phosphorus, TSH, and T4 values. When the absence of PTH resistance is confirmed, a genetic study may be requested to confirm the mutation [1,3]. Treatment of PHP involves oral calcium supplementation, calcitriol, and dietary phosphate restriction. Hypothyroidism may be treated with levothyroxine. Patients may develop complications such as basal ganglia and subcortical white matter calcification, carpal tunnel syndrome, impaired glucose tolerance, spinal stenosis, and movement limitation [1]. Besides these, patients with AHO may develop complications such as subcutaneous calcifications, obesity, and development delay [1].

As seen in the literature, the case presented describes a patient with the clinical characteristics of AHO (short stature and neurocognitive impairment) who presented with an acute disease to the ED. During hospitalization, these clinical features were noted, and, in conjunction with typical ion alterations (severe hypocalcemia and hyperphosphatemia), an etiological investigation was done. Due to the limitation in the hospital’s lab, PTH and *GNAS* gene studies were only obtained after. The PTH values were normal so the diagnosis was only fully confirmed with the gene mutation result. This case stresses the importance of not only treating the acute disease but also looking at the patient and their clinical and analytical features to diagnose this disease, as the patient went 55 years without diagnosis. As AHO may lead to various complications, related to its own phenotype and associated diseases, its early diagnosis may prevent disease progression and comorbidities.

## Conclusions

AHO is a rare disease caused by a gene mutation. Practitioners must consider this disease when patients present with a range of specific clinical features in association with metabolic impairment of calcium and phosphorus and the absence of PTH resistance. Treatment options are limited, as only supportive measures are available, with supplementation and dietary restrictions. As PHP is rare, it is crucial to conjugate clinical and analytical features to not ignore this disease and prevent its complications.
